# Retraction: Upregulation of miR-26b alleviates morphine tolerance by inhibiting BDNF *via* Wnt/β-catenin pathway in rats

**DOI:** 10.1039/d1ra90020j

**Published:** 2021-01-20

**Authors:** Laura Fisher

**Affiliations:** Royal Society of Chemistry Thomas Graham House, Science Park, Milton Road Cambridge CB4 0WF UK advances-rsc@rsc.org

## Abstract

Retraction of ‘Upregulation of miR-26b alleviates morphine tolerance by inhibiting BDNF *via* Wnt/β-catenin pathway in rats’ by Xing Liu *et al.*, *RSC Adv.*, 2019, **9**, 40895–40902, DOI: 10.1039/C9RA06264E.

The Royal Society of Chemistry hereby wholly retracts this *RSC Advances* article due to concerns with the reliability of the data. The images in the article, and the raw data provided by the authors, were screened by an image integrity expert.

All the western blot panels are over-contrasted, have no background and do not look genuine. The raw data provided by the authors has been digitally cropped, and there is evidence of manipulation in the backgrounds, therefore the bands have likely been pasted onto false backgrounds. The expert was able to identify cloned sites in the raw data for multiple panels including Fig. 1C (bottom 3 panels), Fig. 5A (β-catenin) and Fig. 6A (all panels), indicating that the raw data was manipulated. Therefore, the raw data provided by the authors cannot be used to validate the published data.

Given the significance of the concerns about the validity of both the data in the article and the raw data provided by the authors, the findings presented in this paper are not reliable.

The authors have been informed but have not responded to any correspondence regarding the retraction.

Signed: Laura Fisher, Executive Editor, *RSC Advances*

Date: 7^th^ January 2021

## Supplementary Material

